# Single Accreditation and The Match: How Does the Change Affect Osteopathic Graduates?

**DOI:** 10.15694/mep.2020.000066.1

**Published:** 2020-04-08

**Authors:** Randall L. Culbertson, Samuel Kadavakollu, Boris Boyanovsky

**Affiliations:** 1 California Health Sciences University

**Keywords:** Graduate Medical Education (GME), Osteopathic recognition, Single accreditation system, The Match

## Abstract

This article was migrated. The article was marked as recommended.

There is a predicted serious shortage of physicians in the US. To overcome this problem, medical schools have expanded at the behest of the US Government by number and by overall graduates - both medical doctors and doctors of osteopathic medicine. However, the graduate medical program numbers are struggling to keep pace with the significant additional medical school graduates to adequately accommodate the larger supply of applicants for GME positions. Moreover, 2020 marks the first single accreditation system for graduate GME in the United States. Although a historical moment, it further contributes to the uncertainty of GME program availability and medical graduates match rates. In this article, we make a thorough analysis of traditional and recent matching rates for the American Association of Colleges of Osteopathic Medicine and the Accreditation Council for Graduate Medical Education programs for osteopathic medical graduates compared to medical school graduates and provide an insight of the 2020 single accreditation system match.

Our data indicate that osteopathic medical graduates show increasing matching rates to previously ACGME programs, and the 2020 match shows the best matching rate so far.

## Introduction

There is a predicted shortage of 122,000 physicians by the year 2032, including up to 55,000 primary care physicians in the US ( Petterson *et al.*, 2012). To cope with this huge demand, US medical school enrollment has grown by 31% in 2019, according to the medical school enrollment survey report released recently by the American Association of Medical Colleges ( Janis Orlowski, 2019). Similarly, the overall first-year medical school enrollment in “doctor of osteopathy” (DO) - granting schools combined with schools, granting “medical doctor” (MD) degree has grown by 52% since 2002 ( Janis Orlowski, 2019). However, the availability of graduate medical education (GME) opportunities at the national level have not expanded proportionally, representing another significant problem in the medical community. At this point, the US GME needs an adequate number of residency programs to accommodate the increased number of medical graduates.

Another factor that will considerably influence the residency programs is the American Association of Colleges of Osteopathic Medicine (AACOM) and the Accreditation Council for Graduate Medical Education (ACGME) “merger.” Recently, the American Osteopathic Association (AOA), the AACOM, and ACGME reached a historic agreement to establish a single accreditation system for graduate GME in the United States ( Buser *et al.*, 2018). The transition period started in 2015 and will end this year. Therefore, it is imperative to start thinking about the challenges this change will impose ( Cummings, 2017). In this article, we discuss the single accreditation, the residency program match, and the effects of the AACOM and ACGME merger on osteopathic medical students.

The 2019 spring match was the last AOA match before a single accreditation process started. This translates to the days of essentially 100% matching for DO graduates to AOA programs are over, and the match will be affected by MD graduates and/or Foreign Medical Graduates. The most essential question therefore is: does the single accreditation offer increased opportunities for training of osteopathic medicine or not? It appears that it does for MD graduates due to the fact that they may apply for osteopathic recognition, a distinction that identifies their commitment to teaching osteopathic principles and practices.

## Reflections of Match Data

The National Resident Matching Program (NRMP) started in 1952, optimizes the rank-ordered choices of applicants to program directors. Surprisingly, the first match was conducted for 6,000 graduating U.S. medical school seniors when 10,400 internship positions were available. In 2019, the number of registrants reached to 44,603 for the total number of 35,185 available positions. This clearly explains the increasing trend in the total number of applicants and the suboptimal number of residency programs. The overall NRMP match rate since 2009 is 78.3%, with the US allopathic and osteopathic graduates leading the match rates by a substantial margin, compared to international medical graduates (IMGs) and others.

For the last five years, AOA programs that desired to continue their existence have been feverishly working on applications and sponsoring institution agreements to be initially accredited through ACGME. Most have succeeded, and only a small number were not able to receive initial accreditation and were closed. In 2018, 918 AOA programs (75%), had submitted the application for accreditation through ACGME. Of those, 278 AOA programs (30%) achieved initial accreditation, and 53 (6%) closed ( Monir, 2020). As of March 2019, 66% of all AOA programs existing in 2015 have achieved initial accreditation, and 6% are still in pre-accreditation. Residency programs in underserved areas sponsored by Federally Qualified Healthcare Centers called Teaching Health Centers have done well in achieving initial accreditation reaching 85% approval.

The loss of traditionally DO residencies is most obvious in the surgical specialties where the traditional AOA programs have been in community hospitals and not in large academic medical centers, which has been standard for the ACGME programs. For example, in 2015, the AOA match had 3,118 positions available in 774 programs. Of those, 939 seats were unfilled. In general surgery, there were 149 positions, and in orthopedic surgery, there were 111 positions available for DO graduates. In contrast, in the final 2019 AOA match, there were 1,276 positions available in 347 programs, and 390 positions remained unfilled. General surgery dropped to 74 positions and orthopedics to 109. This was not a surprise since this was the last match for the AOA and all residencies starting in 2020 would be affiliated with the ACGME. Also, there is a substantial growth of DO graduates in the match since 2009.

**Table 1.** Percentage match rate by specialty between medical school (MD) graduates and osteopathic medical school graduates (DO)

Specialty	MD graduates	DO graduates
Anesthesiology	96%	90%
Diagnostic Radiology	89%	81%
Emergency Medicine	89%	81%
Family Medicine	90%	95%
Internal Medicine	98%	92%
IM/Peds	93%	85%
Neurology	96%	91%
Pathology	96%	93%
Pediatrics	99%	88%
Physical Medicine	88%	66%
**OB-GYN**	**88%**	**61%**
**General Surgery**	**84%**	**50%**
**Dermatology**	**81%**	**39%**
**Neurosurgery**	**86%**	**30%**
**Orthopedic Surgery**	**82%**	**23%**

For many years, family practice and internal medicine programs have been open to a significant number of DO graduates. Thus, DO graduates are unlikely to suffer any significant loss of opportunity in primary care residencies. This is unlikely to be the story in surgical specialties and other “highly sought-after” programs, where DO graduates have had limited success. Examples are dermatology, radiology, in addition to the surgical programs. This shows that we need to increase the number of ACGME accredited former AOA programs and hope that now, dually certified programs will continue to offer OPP teaching through the osteopathic recognition pathway. There will also be an opportunity to offer osteopathic recognition to many more programs in a variety of specialties. Comparing the traditional match rates for the graduates of allopathic and osteopathic residency by specialty, we can see that highly sought-after programs, especially in surgical specialties and dermatology, have low osteopathic students match rates compared to allopathic students ( Table 1).

We can easily see that traditionally the rate for MD and DO graduates matching is similar in primary care areas and other specific special+ties like Neurology. However, in surgical specialties like Neurosurgery, General Surgery, and Orthopedic Surgery, the margins are extremely wide.

This disparity will take time to overcome. Also, the AOA still had a match in 2018 and 2019 where DO’s in these specialties may have matched. Eventually, the future matches will tell a much more accurate story ( NRMP, 2018).

We know that with single accreditation, there will be an equal opportunity for MD and DO students to match in all specialties. We don’t have enough data to look at otolaryngology, ophthalmology, and urology at this time, and a few years of data are necessary to draw valid conclusions.

The single accreditation match has been a significant success despite early fears. Many changes have occurred since the first announcement in 2014. For example, all AOA board-certified physicians are qualified to be program directors for any ACGME program. Initial resistance in some surgical specialties, in particular, has slowly dissipated. Despite the significant progress, it may take time to realize where we are with regard to the selection, especially in heavily sought-after programs like Ophthalmology, Dermatology, Orthopedic Surgery, General Surgery, and Neurological Surgery.

This issue could be resolved faster if there was a better way for program directors to understand the COMLEX versus USMLE scores. We would propose that the raw score for each exam to be compared as a percentile among medical students who have taken both board scores. Providing this data, the 80 ^th^ percentile in COMLEX could be fairly compared to and 80 ^th^ percentile score on the USMLE. The raw scores may not be as well understood as a percentile score.

PGY-1 positions are increasing, thank goodness, although not keeping pace with the substantial increase of medical students graduating from so many new or expanding medical schools. In 2017, there were 28,849 PGY-1 positions in all specialties. In 2019 ( Porter, 2019), that number has risen to 32,194. This is good news. We know from 2019 that 93.9% of MDs obtained a GME position in the Match and 84.6% of DO seniors matched in the NRMP Match. As a comparison, the match rate for 2019 US MD seniors was 93.9%. A total of 100 DO students did not match or place into GME, while 623 US MD students did not obtain GME positions even after the SOAP in 2019.

In addition, the current DO’s specialty distribution nationwide is as follows: 38% Family practice, 18% Internal medicine, 8% Emergency medicine, 5% Pediatrics, and 5% OB-GYN. These are followed closely by 4% Surgery, 4% Anesthesiology, 4% Neurology/Psychiatry, and 3% Orthopedic surgery. Distribution for positions among MD and DO graduates were as follows: Of the 8,116 Internal medicine positions offered, 41.5% (n=3,366) went to MDs, and 14.8% (n=1202) went to DOs. Of the 4,107 Family Medicine positions offered, 39% (n=1,601) went to MDs and 24% (n=986) went to DOs. In pediatrics 2,847 positions were offered with 60% (n=1,715) going to MDs and 17.6% (n=502) to DOs (NRMP DATA). The represented data do not include positions occupied by IMGs.

## The 2020 Match; does data indicate we met or exceeded expectations for osteopathic graduates?

It is important to compare the data from 2019 to the first single accreditation year of 2020 to see if there is continued progress and predict the future to a higher degree. Based on the conclusions in 2019 above, we have decided to review the results from the first single accreditation match of 2020.

As a background, let’s look at some key statistics from 2019. Enrollment and the number of osteopathic medical schools have climbed consistently. Just since 2017, we have moved from 27,485 students in 44 schools to 30,918 students in 49 schools. In 2020 two new schools will open, followed by more openings the next year and so on. Osteopathic students make up just over 25% of all US medicals students currently, and the percentage is climbing yearly at a rate near 5% per year.

The 2020 Match results are now available ( NRMP, 2020). These exciting results now tell us more about how DO graduates will compare with US MD students and IMGs for residency positions.

The 2020 Match is the largest in NRMP history with a record of 40,084 applications submitted for 37,256 positions. The number of PGY-1 positions was a record high of 34,266, which was an increase of 2,072 over 2019 (6.4%). This was expected as all DO graduates were required to enter the single accreditation system. The increased number of PGY-1 spots has been a requirement of all new DO schools, which have been increasing significantly over the last ten years. There has also been a push from new MD schools to also work to increase the number of GME spots especially PGY-1 spots. Primary care residency positions increased by 581 in Internal Medicine, 555 in Family Medicine, and 17 in Pediatrics.

Finally, for the 2020 match, the totals of MD seniors who entered the match was 19,326 and 18,108 matched (93.7% match rate). The total of DO seniors who entered the match was 6,581 and 5,968 matched (90.7% match rate). The percentage of DO school seniors was 25% of all medical graduates, and 75% were MD seniors. The 2020 match data clearly show that the DO matching rate (90.7%) has substantially increased compared to the DO match to ACGME programs in 2019 (80.2%). Table 2 shows some examples of data regarding specialty success for US MD seniors and US DO seniors competing for PGY-1 slots in 2020, which are discussed in the next paragraph.

**Table 2.** Distribution of medical specialties between MD and DO seniors

Specialty	MD Seniors 19,326	DO Seniors 6,581
Family medicine	1,543 (8.0%)	1,392 (21.2%)
Emergency medicine	1,713 (8.9%)	683 (10.4%)
Internal Medicine	3,496 (18.1%)	1,389 (21.1%)
Neurological Surgery	232 (1.2%)	3 (0.05%)
Orthopedic Surgery	686 (3.6%)	112 (1.7%)
OB-GYN	1,089 (5.6%)	221 (3.4%)
Surgery	1,033 (5.3%)	202 (3.0%)

## Conclusions and Future Directions

The results are, in my view, better than we would have anticipated five years ago in primary care specialties. Although at first glance, for programs like Neurological surgery, orthopedic surgery, OB/GYN, and surgery, DO graduates match at lower numbers, if calculated as percent from the total MD or DO pool of applicants, it becomes obvious that the match rates are comparable. Overall, this was a very good first single accreditation match for the osteopathic profession. However, more work needs to be done to reach equivalent levels in surgical and highly sought-after programs. The USMLE scores still tend to be used in these programs to a much greater extent, while primary care specialties seem to be applying more appropriate other criteria in choosing their residents much as medical schools are for choosing which applicants are selected. I think this will change with time as old habits are hard to break.

The osteopathic profession is growing not only in numbers but in stature. The holistic and wellness tenets of DO education for many years are now key aspects of allopathic education. Many MDs were becoming more and more interested in neuromuscular medicine and started understanding osteopathic principles. We believe the structure and demands of the ACGME are continuing to improve graduate medical education, and the osteopathic profession is in the middle of this effort just as they should be. The key issue which we will need to address through congressional help is still an expansion of GME positions. As we discussed, this year, 40,084 applicants submitted program choices for 37,256 positions. This number also includes foreign medical grads. There were 5,167 US citizen foreign medical grads seeking residency and 6,907 non-US citizen foreign medical grads for a total of 12,074. This shows that currently, we have enough spots to potentially match all US senior graduates from MD and DO schools but not all the foreign medical graduates. The last few matches have shown only about 55-59% of foreign medical graduates were matched successfully.

The match, while it has enough spots for US medical grads, the number of spots in some desired specialty may not be meeting the need. We still need to take a comprehensive look at current GME programs from and number and type perspective and determine where we can add positions. There are many hospitals with the desire and capability to have good quality programs that are stuck with years old caps and zero PRA’s which prevent them from adding positions in a cost-effective manner. We need congressional help to allow these hospitals with zero per resident amount and a very small cap under 5 to re-establish a program and build needed GME positions. While we have apparently combined osteopathic and allopathic residency training under one body, there is still more work to be done.

## Take Home Messages

• The overall residency program positions increase at a suboptimal level compared to the largely increasing number of medical graduates.
• The primary care programs are still predominantly represented by DO physicians.
• Although surgical specialties are still represented by DO physicians at a lower percentage, it appears that the single accreditation closes the previously observed wide margin between DO and MD physicians.
• The single accreditation program appears to mutually benefit MD and DO physicians.

## Notes On Contributors

**Randall L. Culbertson** DO, MBA, is an Associate Dean for Graduate Medical Education at The California Health Sciences University College of Osteopathic Medicine.

**Samuel Kadavakollu** Ph.D. is an Associate Professor of Biomedical Education and Director of MCAT and Preparatory Programs at The California Health Sciences University College of Osteopathic Medicine.

**Boris Boyanovsky** MD, Ph.D. is an Associate Professor of Biomedical Education at The California Health Sciences University College of Osteopathic Medicine.
