# Supplementary material for: Single Accreditation and The Match: How Does the Change Affect Osteopathic Graduates?
Source: MedEdPublish (2016). 2020 Apr 8;9:66. [Version 1] doi: 10.15694/mep.2020.000066.1 (PMC10697474; doi:10.15694/mep.2020.000066.1)
Supplement: Peer review history

# Reviewer report: Reviewer response for version 1

**DOI:** 10.21956/mep.20030.r30603

**Published:** 2020-06-13

**Authors:** Ken Masters

**Affiliations:** 1 Sultan Qaboos University

This review has been migrated. The reviewer awarded 3 stars out of 5 An interesting piece on the impact of single accreditation on Osteopathic graduatesOverall, the piece is very well written. Although it is billed as an opinion piece, the authors have presented a great deal of information to show that their opinion on the topic is solidly grounded in the data. I do, however, have one criticism: the paper has a high number of statistics drawn from elsewhere with almost no clear citations. These statistics require citation. (The authors have supplied references, but the in-text citations are required).So, my low score may come as a disappointment to the authors, but, these citations really are necessary. I look forward to a Version 2 of this paper, so that I can re-review it with an improved rating.

Reviewer Expertise:

NA

No decision status is available

# Reviewer report: Reviewer response for version 1

**DOI:** 10.21956/mep.20030.r30602

**Published:** 2020-04-16

**Authors:** P Ravi Shankar

**Affiliations:** 1 American International Medical University

This review has been migrated. The reviewer awarded 4 stars out of 5 This is an interesting article which introduced me to osteopathic medical schools and the single accreditation program for graduate medical education. The article is well-written and the authors present their ideas using a logical framework. The article provides insights about residency matching, the shortage of physicians in the United States and increasing intakes at US medical schools and also increasing number of residency positions.

Reviewer Expertise:

NA

No decision status is available
